# Throat Swabs and Sputum Culture as Predictors of *P*. *aeruginosa* or *S*. *aureus* Lung Colonization in Adult Cystic Fibrosis Patients

**DOI:** 10.1371/journal.pone.0164232

**Published:** 2016-10-06

**Authors:** Darius Seidler, Mary Griffin, Amanda Nymon, Katja Koeppen, Alix Ashare

**Affiliations:** 1 Pulmonary and Critical Care Medicine, Dartmouth-Hitchcock Medical Center, Lebanon, NH, United States of America; 2 Microbiology and Immunology, Geisel School of Medicine at Dartmouth, Hanover, NH, United States of America; Laurentian, CANADA

## Abstract

**Background:**

Due to frequent infections in cystic fibrosis (CF) patients, repeated respiratory cultures are obtained to inform treatment. When patients are unable to expectorate sputum, clinicians obtain throat swabs as a surrogate for lower respiratory cultures. There is no clear data in adult subjects demonstrating the adequacy of throat swabs as a surrogate for sputum or BAL. Our study was designed to determine the utility of throat swabs in identifying lung colonization with common organisms in adults with CF.

**Methods:**

Adult CF subjects (n = 20) underwent bronchoscopy with BAL. Prior to bronchoscopy, a throat swab was obtained. A sputum sample was obtained from subjects who were able to spontaneously expectorate. All samples were sent for standard microbiology culture.

**Results:**

Using BAL as the gold standard, we found the positive predictive value for *Pseudomonas aeruginosa* to be 100% in both sputum and throat swab compared to BAL. However, the negative predictive value for *P*. *aeruginosa* was 60% and 50% in sputum and throat swab, respectively. Conversely, the positive predictive value for *Staphylococcus aureus* was 57% in sputum and only 41% in throat swab and the negative predictive value of *S*. *aureus* was 100% in sputum and throat swab compared to BAL.

**Conclusions:**

Our data show that positive sputum and throat culture findings of *P*. *aeruginosa* reflect results found on BAL fluid analysis, suggesting these are reasonable surrogates to determine lung colonization with *P*. *aeruginosa*. However, sputum and throat culture findings of *S*. *aureus* do not appear to reflect *S*. *aureus* colonization of the lung.

## Introduction

Patients with cystic fibrosis (CF) are plagued by chronic respiratory infections and inflammation leading to progressive bronchiectasis and, ultimately, respiratory failure. Pulmonary disease is the leading cause of morbidity and mortality in CF [1]. The prevalence of each microbial species varies with age. The most prevalent species are *Pseudomonas aeruginosa* and *Staphylococcus aureus*. *P*. *aeruginosa* is recognized as the main pathogen responsible for the morbidity and mortality of CF patients [2–4]. The prevalence of *P*. *aeruginosa* infection increases with age and more than 80% of patients older than 19 years are chronically infected [2, 4–6]. Chronic infections with *S*. *aureus*, both methicillin sensitive (MSSA) and methicillin resistant (MRSA) are also on the rise for the CF population as a whole [4]. Early identification of *S*. *aureus* is important, as MRSA is associated with an adverse outcome [7, 8]. Therefore, an accurate assessment of lower respiratory tract colonization is critical to inform accurate therapy.

Quarterly respiratory cultures are obtained as standard of care to inform treatment, a practice based on non-evidence based guidelines [4, 9]. Though sputum is preferred over throat culture [10], not all patients are able to spontaneously produce a sputum sample. In fact only 35–40% of pediatric patients produce sufficient sputum to be sent for culture [11, 12]. When patients are unable to produce a sputum sample, clinicians often obtain throat swabs and send them for culture as a surrogate for lower respiratory tract cultures. This practice may be useful in subjects not known to be colonized with *P*. *aeruginosa* in order to catch asymptomatic patients with first time colonization, but its clinical utility in the adult CF population is unclear [9, 13]. In fact Armstrong et al. argue that oropharyngeal cultures are poor predictors of pathogens in children with CF [14]. As it stands we don’t know how accurate sputum cultures and throat swabs are compared to the gold standard of bronchoalveolar lavage (BAL). Our aim in this study is to determine the reliability of throat cultures and sputum cultures.

Despite arguments against throat swabs as standard of care in children who cannot produce sputum, others found that throat swabs show excellent concordance with cultures obtained from a bronchoalveolar lavage [11]. In addition, in children <18 months Rosenfeld et al found that compared to BAL, oropharyngeal cultures for *P*. *aeruginosa* yield a sensitivity of 44%, specificity of 95%, positive predictive value (PPV) of 44% and negative predictive value (NPV) of 95% [15]. Ramsey et al report a PPV of 83% for *P*. *aeruginosa* and a PPV of 91% for *S*. *aureus* in non-expectorating subjects <10 years of age [11]. To complicate matters, throat swabs may underestimate bacteria associated with airway inflammation in subjects age 8–21 years [13]. While there are ample data describing the relationship between throat swab and BAL cultures in children, there are minimal data looking at the predictive value of throat swabs and sputum in adult patients with CF. In this study, we sought to determine the positive and negative predictive value of throat and sputum culture for the most common CF organisms.

## Materials and Methods

### Human Subjects

This study was approved by the Committee for the Protection of Human Subjects at the Geisel School of Medicine at Dartmouth. Following written informed consent, subjects underwent a throat swab, which was sent for standard CF culture. Throat swab samples were obtained by the same provider in all subjects by swabbing the posterior oropharynx. If the subject was able to spontaneously expectorate, a sputum sample was also sent for culture. Subsequently, flexible bronchoscopy was performed. Briefly, a bronchoscope was passed through the vocal cords after local anesthesia with viscous lidocaine to the posterior pharynx and intravenous sedation. No suction was used prior to obtaining the BAL sample to ensure that the suction channel was not contaminated. BAL fluid was obtained from the anterior segment of the right upper lobe (RUL) and sent for culture. In all subjects, 20cc of sterile saline was inserted into the RUL and aspirated back into a sterile container. Following the procedure, subjects were monitored until they were stable for discharge. In a subset of subjects (n = 3), two bronchoscopes were used and the first bronchoscope was inserted to just above the vocal cord and the channel was washed with normal saline and cultured to assess for oral contamination during the bronchoscopy procedure. Adult CF Subjects (n = 20) were enrolled if they were not currently having an exacerbation and were non-smokers. While subjects could be on a chronic inhaled antibiotic regimen, all subjects had been off antibiotics for exacerbation treatment for at least 28 days. All female subjects underwent a pregnancy test and were excluded if positive.

### Microbiology Culture

Specimens of expectorated sputum, BAL, and deep throat swabs were processed following the recommendations of the Microbiology Working Group of the Northern New England Cystic Fibrosis Consortium and the Microbiology Consensus Conference of the Cystic Fibrosis Foundation [4]. Gram stains were performed on all specimens and no specimen was rejected because of upper respiratory contamination. The most purulent portion of a specimen was identified, inoculated onto agar plates by swab and streaked for isolation of colonies. Media inoculated were 5% sheep blood agar, chocolate agar, colistin/nalidixic acid agar, MacConkey agar, mannitol-salt agar, and oxidation-fermentation polymyxin bacitracin lactose agar (all from Remel, Lenexa Kansas). Agar plates were incubated at 35 degrees centigrade for a minimum of 72 hours, and observed for growth of pathogens specified by the CF Consensus document, including *Staphylococcus aureus*, beta-hemolytic *streptococci*, *Streptococcus pneumoniae*, *Streptococcus milleri* group, *Haemophilus influenza*, *Moraxella catarrhalis*, *Pseudomonas aeruginosa*, *Stenotrophomonas maltophilia*, *Achromobacter secies*, *Acinetobacter species*, *Burkholderia* species and yeast.

### Statistical Analyses

Sample size calculation was determined using G*Power Statistical Software. Using a Fisher’s Exact Test and setting the phi coefficient at 0.7 (strong positive association), we would need to enroll 20 subjects to detect a positive association with a power of 90%. Statistical analyses of data were performed using GraphPad Prism 6 for Mac OS X software version 6.0h (Prism, San Diego, CA) and the R software environment for statistical computing and graphics [16].

## Results

### Patient Characteristics

A total of 20 subjects with CF were enrolled in this study. The demographic and genetic attributes of this cohort are listed in **Table 1**. The median age of the cohort was 27 years. All subjects possessed at least one F508del-CFTR allele.

**Table 1:** Patient Characteristics.

Subject	Age (y)	Gender	FEV1 (% predicted)	Sputum PA	Sputum Staph	Throat PA	Throat Staph	BAL PA	BAL Staph
1	25	M	64%	Y	Y	Y	Y	Y	N
2	28	M	117%	Y	N	Y	Y	Y	N
3	28	M	88%	ND	ND	N	Y	N	Y
4	31	M	90%	ND	ND	N	Y	Y	N
5	24	M	60%	N	Y	N	Y	Y	Y
6	24	M	84%	N	Y	N	Y	N	N
7	25	F	85%	Y	N	Y	Y	Y	N
8	27	M	72%	Y	N	Y	N	Y	N
9	34	F	56%	Y	N	Y	N	Y	N
10	21	M	92%	N	Y	N	Y	N	Y
11	36	M	106%	ND	ND	N	Y	Y	Y
12	28	F	82%	ND	ND	Y	Y	Y	N
13	22	F	65%	Y	Y	N	Y	Y	Y
14	21	F	86%	N	Y	N	Y	Y	N
15	30	M	73%	N	Y	N	Y	N	Y
16	19	M	116%	ND	ND	N	Y	N	N
17	53	M	74%	ND	ND	N	Y	N	N
18	20	F	63%	ND	ND	N	Y	N	N
19	37	M	94%	ND	ND	N	Y	Y	Y
20	26	M	51%	Y	N	N	N	Y	N

PA: Pseudomonas aeruginosa; Staph: *Staphylococcus aureus*; ND: not done

### Microbiological Relationship between BAL Fluid Culture, Sputum Culture, and Throat Culture

BAL fluid culture and throat culture were obtained from 20 subjects with CF. Cultures were processed via standard methodology at the DHMC Microbiology Laboratory and within the guidelines of the Cystic Fibrosis Foundation [4]. In all cases, the throat swab was obtained immediately prior to the bronchoscopy. A subset of subjects (n = 12) also had a spontaneously expectorated sputum sample obtained for culture on the same day and prior to the bronchoscopy.

To assess for contamination of BAL fluid by oral flora, in a subset of subjects (n = 3) a scope was with normal saline was performed before the bronchoscope was inserted past the vocal cord. In 2 subjects, standard culture revealed only “normal oral flora” in the scope wash and did not demonstrate a predominant organism. In the third subject, no bacteria were isolated from the scope wash. These data suggest that the culture results obtained in BAL fluid do not reflect contamination of the bronchoscope by oral flora.

We calculated specificity, sensitivity, positive predictive value (PPV) and negative predictive value (NPV) of throat culture results for the two most common CF pathogens as identified by Cystic Fibrosis Foundation registry data using BAL culture results as the gold standard. Comparing sputum to BAL for *P*. *aeruginosa*, we found a sensitivity of 78%, specificity of 100%, PPV of 100%, and NPV of 60%. For *S*. *aureus* found in sputum versus BAL we calculated a sensitivity of 100%, specificity of 63%, PPV of 57%, and NPV of 100%. Analyzing throat swab *P*. *aeruginosa* compared to BAL, we found a 46% sensitivity, and 100% specificity yielding a PPV of 100% and NPV of 50%. Throat swab compared to BAL for *S*. *aureus* showed a sensitivity of 100%, specificity of 23%, PPV of 41%, and NPV of 100%.

**Fig 1:**
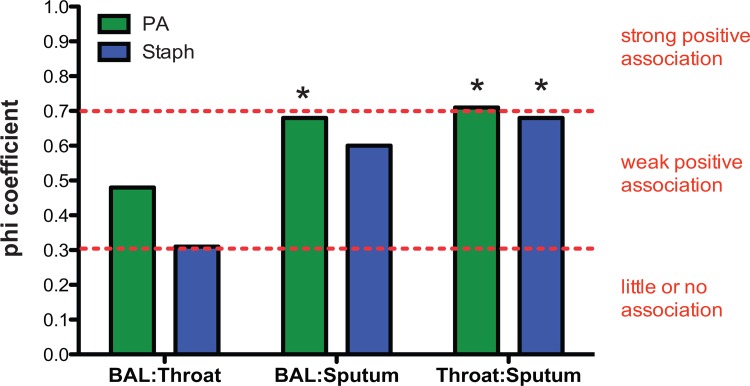
Comparison of Methods for Determining Bacterial Colonization. Clinical samples were obtained from CF subjects (n = 20 for BAL and throat swab and n = 12 for sputum). The phi coefficient was used to measure the degree of association between positive and negative cultures for *P*. *aeruginosa* and *S*. *aureus* in BAL fluid, sputum and throat swabs. Throat swabs and sputum were significantly correlated for both pathogens, as were BAL fluid and sputum for *P*. *aeruginosa* (p < 0.05 as determined with Fisher’s Exact Test and indicated by an asterisk).

We calculated the phi coefficient to measure the degree of association between BAL and throat culture, BAL and sputum culture or throat and sputum culture for both *P*. *aeruginosa* and *S*. *aureus* (**Fig 1**). A phi coefficient above 0.7 signifies a strong positive association, between 0.7 and 0.3 indicates a weak positive association and below 0.3 suggests little or no association. The correlations between *P*. *aeruginosa* BAL and sputum culture and throat and sputum culture as well as *S*. *aureus* throat and sputum culture were statistically significant as determined by Fisher’s Exact Test (p < 0.05). Meanwhile, the correlations between *S*. *aureus* BAL and sputum culture and BAL and throat cultures for both pathogens failed to reach statistical significance.

## Discussion

Our data demonstrate that positive sputum and throat culture findings of *P*. *aeruginosa* reflect results found in BAL fluid analysis, suggesting these are reasonable surrogates to determine lung colonization with *P*. *aeruginosa*. The diagnostic challenge lies within those subjects that have a negative throat swab or sputum culture as we cannot rely on this to rule out lung colonization as our data found a low negative predictive value for *P*. *aeruginosa* in both sputum and throat cultures. In adult subjects with CF, sputum and throat culture findings of *S*. *aureus* do not appear to reflect *S*. *aureus* colonization of the lung. However, a negative throat swab for *S*.*aureus* suggests that the organism is likely not present at all. This is very intuitive as *S*. *aureus* is known to colonize the upper airways but this does not necessarily reflect lung colonization.

Obtaining a “standard” culture may lead to identification of a particular organism, but may be subject to differences in competitive growth patterns *in vitro* versus *in vivo*. A weakness of our study is the use of conventional culture without using deep sequencing techniques. 16S ribosomal RNA (rRNA) gene sequencing allows for detection of a larger number of potential pathogens. Only a small fraction of all bacteria can be successfully cultured, while clinically significant organisms may be slow-growing, fastidious, inert, or unviable [17, 18]. Standard culture typically only shows the most abundant organism(s), whereas DNA based techniques can detect minor species, which may be just as important in influencing the pathogenicity and potential inflammatory changes [19, 20]. Consistent with prior studies [21], we recently reported that the sputum microbiome is different from the BAL microbiome in a cohort of adult patients with CF [22]. However, standard clinical practice is to perform quarterly cultures for identification of dominant pathogen(s) so our study sought to investigate how reliable throat and sputum cultures are using the standard methodology. Our future investigations will focus on differences in the microbiome using these more advanced techniques.

Our study was powered to investigate differences in identification of the two most common CF pathogens (*S*. *aureus* and *P*. *aeruginosa*). We recognize that there are many other important pathogens in CF and a potential weakness of our current study was the lack of investigation of these other important bacterial species. However, given that most eradication protocols used clinically are directed at these two most common pathogens [23, 24], early and accurate identification of colonization with *S*. *aureus* and *P*. *aeruginosa* is critical for the effective management of our patients. In addition, despite adequate power, our study is limited by its small sample size and single site. Our future studies will enroll subjects at multiple sites to increase our sample size and allow investigation of geographic differences. Although the microbiome literature suggests stability of the microbiome over time in an individual patient, it is well described that CF patients can be transiently colonized with specific bacteria. A limitation of our study is that it involves a single visit rather than evaluating serial samples.

An important aspect of our study is that it helps the clinician to identify the optimal sampling technique in a clinical context. The predictive value of throat cultures was thoroughly analyzed by Ramsey et al and they found that in non-expectorating patients less than 10 years of age a positive result for *P*. *aeruginosa* was 83% predictive (95% confidence interval 36 to 100%), while *S*. *aureus* was 91% predictive (59 to 100%). Negative throat cultures yielded 70% (48 to 86%) for *P*. *aeruginosa* and 80% (52 to 96%) for *S*. *aureus* [11]. More recently, sputum in children (both induced and spontaneously expectorated) has been compared to throat swab using quantitative PCR concluding that throat swabs may underestimate bacteria associated with airway inflammation [13]. This is consistent with our recent study demonstrating that the microbiome of sputum from CF patients is different from that found in BAL and protected brush samples [22]. Our current data clearly demonstrate that the absence of *P*. *aeruginosa* on throat culture is not adequate to determine if *P*. *aeruginosa* is present in the lung of stable adult CF patients. Further studies using deep sequencing are needed to identify the accuracy of throat and sputum culture in representing the lung microbiome. In addition, studies involving a larger and more diverse patient population will allow for more generalizable findings. If significant differences between BAL and throat swab cultures are found, then the practice of quarterly surveillance cultures using throat swabs as a surrogate for samples obtained from the lung may be challenged.
